# Lipoma arborescens arising in the extra-articular bursa of the knee joint

**DOI:** 10.1051/sicotj/2016019

**Published:** 2016-07-06

**Authors:** Shinji Minami, Yusuke Miyake, Hirofumi Kinoshita

**Affiliations:** 1 Department of Orthopedic Surgery, Naga Municipal Hospital, 1282 Uchita Kinokawa, Wakayama 649-6414 Japan; 2 Department of Orthopedic Surgery, Wakayama Medical University, 811-1 Kimiidera Wakayama 641-8510 Japan

**Keywords:** bursa, extra-articular, knee joint, lipoma arborescens

## Abstract

Lipoma arborescens arising in the extra-articular bursa of the knee joint is extremely rare. We describe an 11-year-old boy who complained of a gradual swelling mass of the lateral knee joint. Magnetic resonance imaging (MRI) showed a high signal intensity tumor on T1- and T2-weighted images with a thickened septa and nodular lesion that showed low signal intensity. The radiologist suggested the possible differential diagnosis of well-differentiated liposarcoma. At operation, the tumor was found under the iliotibial tract and was not in contact with the knee joint. Histopathologically, this lesion was diagnosed as lipoma arborescens arising in the extra-articular bursa of the knee joint. On MRI, the appearance of lipoma arborescens arising in the extra-articular bursa of the knee joint differed from that of conventional intra-articular lipoma arborescens. In this report, we describe a case of extra-articular lipoma arborescens of the knee joint bursa and discuss the diagnosis and etiology.

## Introduction

Lipoma arborescens is a rare synovial lesion, in which the tumor showed diffuse villous proliferation of the synovium characterized by replacement of the subsynovial tissue by mature adipocytes. The most affected site is the knee joint, usually in the suprapatellar pouch of the knee joint [1]. These lesions have been rarely reported in other locations, including the hand, wrist, elbow, shoulder, and hip [2–6], and a few cases have been reported affecting the extra-articular bursa of the deltoid and the tendon sheath [3, 7]; however, extra-articular bursa of the knee joint is thought to be extremely rare. Only one case of lipoma arborescens arising in the extra-articular bursa of the knee joint has been reported in the English literature [8]. On magnetic resonance imaging (MRI), it has been reported that the appearance of conventional lipoma arborescens arising in the knee joint usually shows a villous-like synovial mass with signal intensity similar to that of fat on all sequences and reveals a frond-like villous structure associated with joint effusion [9, 10]; however, our case showed high signal intensity tumor on T1- and T2-weighted images with a thickened septa and nodular lesion that showed low signal intensity. These findings on MRI were similar to those of other lipogenic tumors, including well-differentiated liposarcoma. In this report, we describe a case of extra-articular lipoma arborescens of the knee joint bursa and discuss the diagnosis and etiology.

## Case report

**Figure 1.**
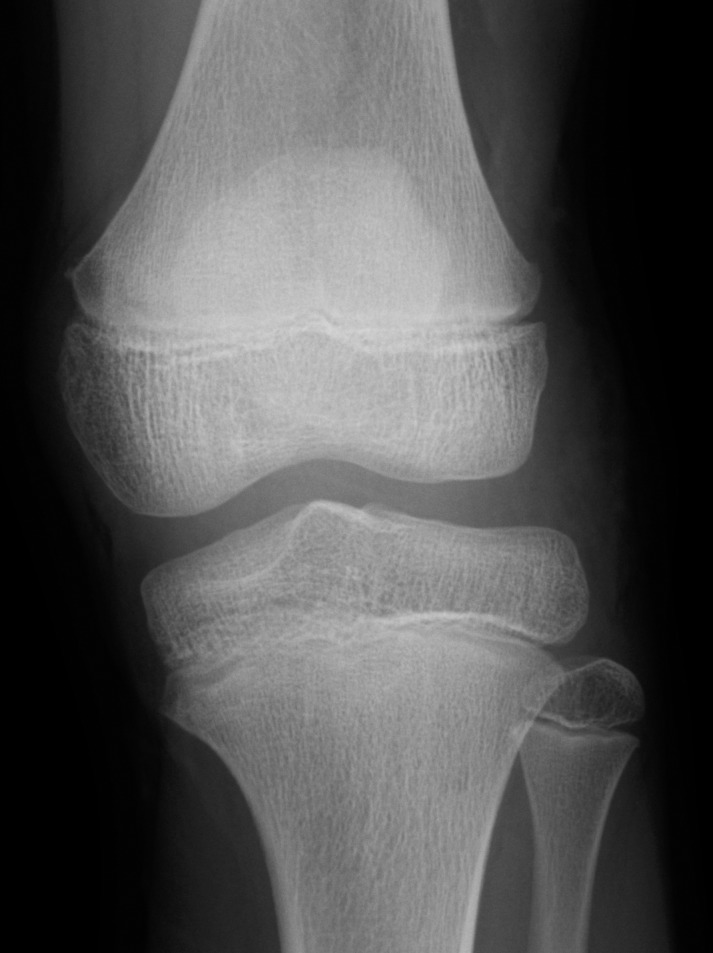
Plain radiographs of the knee joint did not show calcified and osseous lesions.

**Figure 2.**
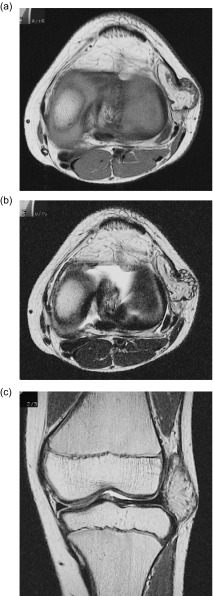
MRI of the tumor demonstrated a high signal intensity area on the T1-weighted image in the axial (a) and T2-weighted image in the axial and coronal views (b, c), and heterogeneously, a low signal intensity area was seen in the tumor. The tumor was located in the extra-capsular lesion of the lateral knee joint and had a diameter of 4 cm.

**Figure 3.**
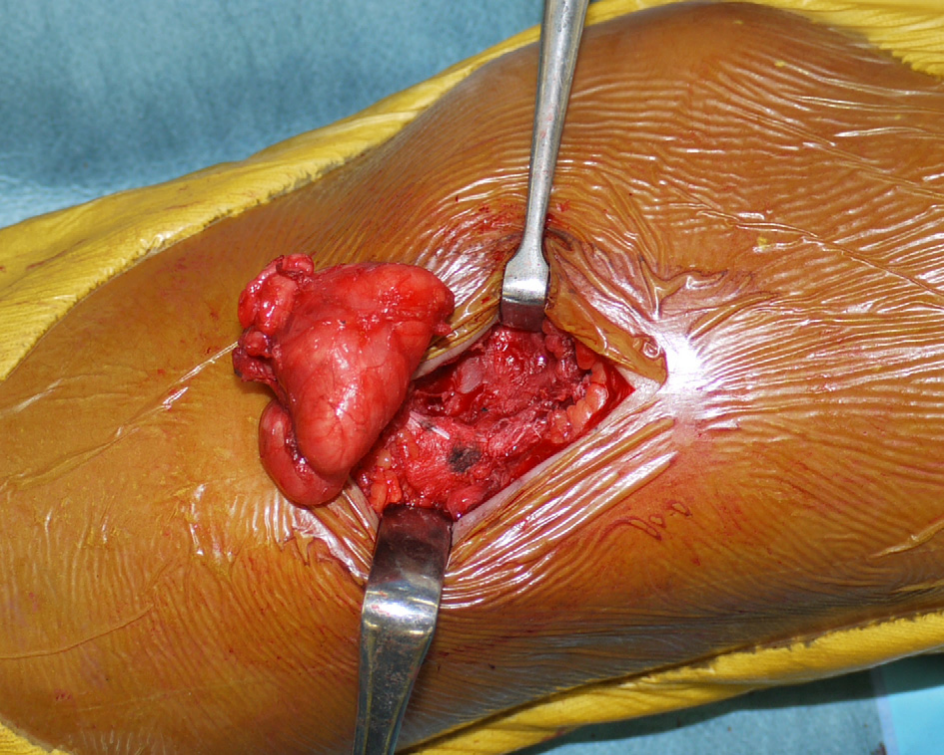
Resection of the tumor. The tumor was a yellow soft mass with a thin capsule. The tumor was found under the iliotibial tract and was not in contact with the knee joint.

**Figure 4.**
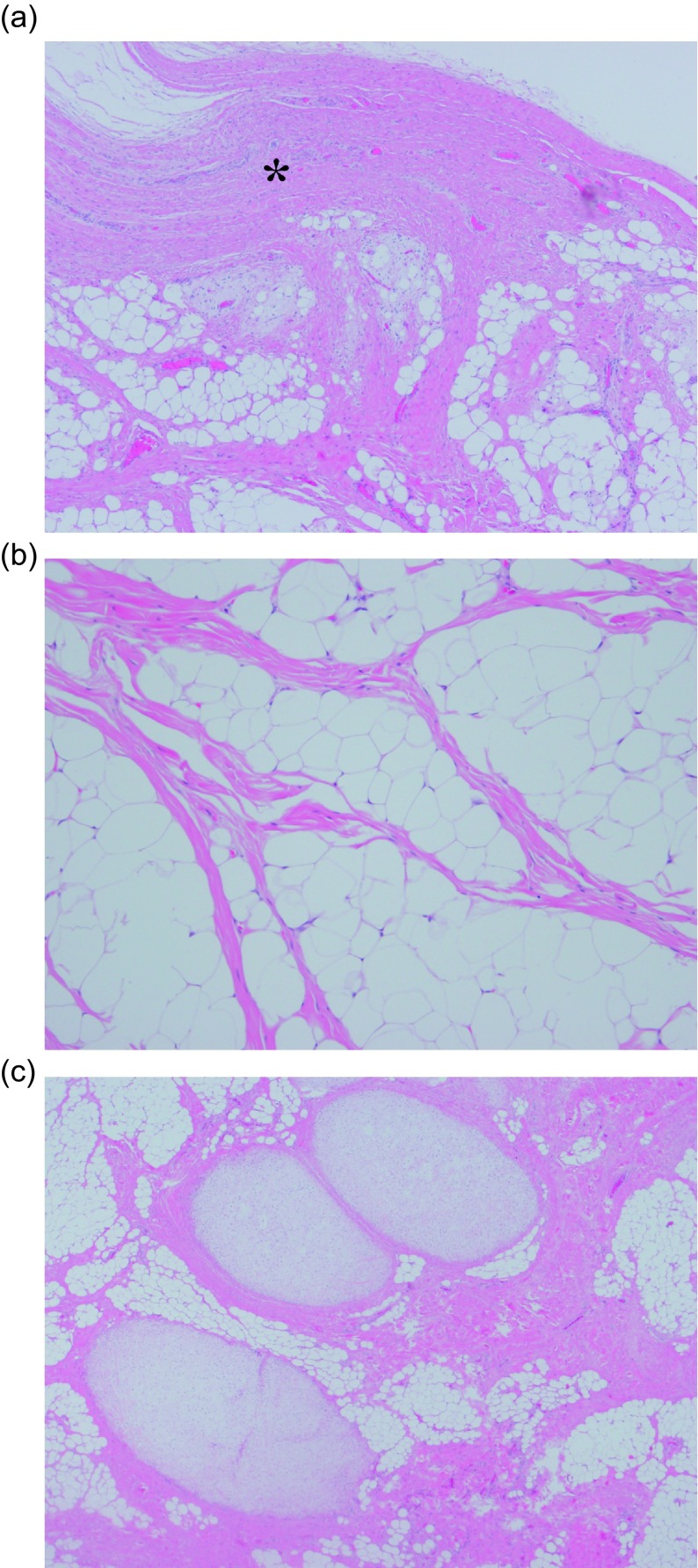
Histopathological examination of excised tumor. (a) Synovial tissue was detected in the margin of this tumor (_*_). In the subsynovia, there was a thick fibrous septum and diffuse proliferation of fat tissue (Hematoxylin and eosin, × 20). (b) Diffuse proliferation of fat tissue was also detected in the tumor and atypical lipoblasts were not seen (Hematoxylin and eosin, × 50). (c) A few chondral nodules and a thick fibrous septa were found in the fat tissue (Hematoxylin and eosin, × 10).

An 11-year-old boy was admitted to our hospital because of swelling and a gradual growing tumor of the lateral knee joint on the left side. He had no history of trauma or infection. The patient’s medical history and the family history were unremarkable, and he played in a football team. Physical examination revealed swelling of the lateral knee joint. The lesion had a normal local temperature and normal skin coloration and was not tender to palpation. The range of motion of the knee joint was normal. Plain radiographs did not show calcified and osseous lesions (Figure 1). MRI demonstrated a high signal intensity area on T1- and T2-weighted images in the axial and coronal views, and a thickened septa and nodular lesion that showed low signal intensity (Figure 2). The tumor had a 4 cm diameter. The radiologist suggested the possible differential diagnosis of well-differentiated liposarcoma. Open biopsy of the tumor was performed. Histopathologically, lipogenic and chondral tissue was detected, but atypical lipoblasts were not seen. Lipogenic tumor with chondral metaplasia and osteochondromatosis were considered for diagnosis. The tumor was resected. At operation, the mass was found under the iliotibial tract and was not in contact with the knee joint (Figure 3). Histopathological examination of the excised tumor showed synovial tissue in the margin of this tumor and, in the subsynovia, diffuse proliferation of fat tissue was also detected with a few chondral nodules and thick fibrous septa. The findings were consistent with chondral metaplasia in lipoma arborescens (Figure 4). This tumor was diagnosed as lipoma arborescens arising in the extra-articular bursa of the knee joint under the iliotibial tract. Informed consent was obtained from the patient for publication of this case report and any accompanying image.

## Discussion

Lipoma arborescens is a rare synovial disorder and the mean age of the reported patients is 43 (range 9–68 years) [11], and pediatric cases are uncommon [12]. Most affected sites of this lesion occur in the knee, especially in the suprapatellar pouch, but extra-articular lesions in the bursa of the deltoid and the tendon sheath are rarely reported. The extra-articular bursa of the knee joint is an extremely rare site. Only one case of lipoma arborescens arising in the extra-articular bursa of the knee joint has been reported [8]. In this report, Kurihashi et al. described that the lipoma arborescens mimicked synovial osteochondromatosis in the lateral knee bursa because the tumor was accompanied with osteochondral metaplasiasa. In our case, histopathological examination also revealed osteochondral tissue in this tumor; therefore, synovial osteochondromatosis could be considered as a differential diagnosis. However, in our case, synovial tissue was also recognized and mature lipogenic tissues in the subsynovia mainly proliferated, but few chondral nodules were detected; therefore, the diagnosis of this tumor was thought to be lipoma arborescens rather than synovial osteochondromatosis. On the other hand, it has been reported that lipoma arborescens on MRI showed a villous frond-like synovial mass with signal intensity similar to that of fat on all sequences [10, 13]. However, these reports mainly explained the appearance of intra-articular lipoma arborescens and it was thought that these findings did not correspond with extra-articular legions. In our case, the tumor mainly showed high signal intensity area on T1- and T2-weighted images in axial and coronal views, and showed a low signal intensity area in the tumor that showed a thickened septa and nodular lesion. At first, the radiologist suggested the possible differential diagnosis of other lipogenic tumors, including well-differentiated liposarcoma. In cases of well-differentiated liposarcoma, the image of a predominantly fatty mass with an irregularly thickened, nodular septa has been reported [14], resembling our case; however, it has been reported that well-differentiated liposarcoma occurs predominantly in middle-aged patients, and atypical lipoblasts are commonly detected. Our case showed diffuse proliferations of fat tissue in the subsynovia and a thick fibrous septum without atypical lipoblasts. Coll et al. reported that the diagnosis of lipoma arborescens recognizes a diffuse synovial origin [15]; therefore, our case could be distinguished from well-differentiated liposarcoma and could be diagnosed as lipoma arborescens. The etiology of lipoma arborescens remains unknown and, as the cause of this lesion, trauma, inflammation, or neoplasm has been postulated [9, 16, 17]. A synovial reaction to a traumatic injury has been proposed [13] but most patients who have lipoma arborescens do not have a history of trauma. In addition, it has been reported that there is an association between lipoma arborescens and degenerative joint disease [18]; however, our patient was too young and degenerative changes and meniscus tears were not observed on MRI. Jaffe suggested that lipoma arborescens represents a non-neoplastic villous synovial proliferation in response to chronic irritation of the synovium [19, 20]. Coll et al. also reported that villous fatty proliferation likely reflects a rare synovial response to chronic irritation, because the lesions were recognized in relatively elderly patients with osteoarthritis [15]. This indicated that chronic irritation of the synovium could be a cause of lipoma arborescens. Although our patient did not show degenerative changes of the knee joint, he played in a football team and therefore vigorously moved his knee joint; therefore, it was thought that chronic mechanical irritation of the extra-articular bursa of the knee joint between the iliotibial tract and the knee joint might have occurred and it could be thought that this was the possible cause of this lesion.

## Conclusion

Lipoma arborescens arising in the extra-articular bursa of the knee joint is extremely rare and, on MRI, the appearance of extra-articular lipoma arborescens is different from that of conventional intra-articular lipoma arborescens. It was considered that extra-articular lipoma arborescens should be diagnosed differentially from synovial osteochondromatosis and other lipogenic tumors, including well-differentiated liposarcoma.

## Conflict of interest

SM, YM, and HK declare no conflict of interest in relation with this paper.
